# Long-term follow-up and exploration of the mechanism of stromal vascular fraction gel in chronic wounds

**DOI:** 10.1186/s13287-023-03389-2

**Published:** 2023-06-19

**Authors:** Yuan Cai, Fang Zhang, Jingwei Feng, Bihua Wu, Hai Li, Shune Xiao, Feng Lu, Zairong Wei, Chengliang Deng

**Affiliations:** 1 Department of Burns and Plastic Surgery, Affiliated Hospital of Zunyi Medical University, 149 Dalian Road, Zunyi, 563000 Guizhou China; 2 The Collaborative Innovation Center of Tissue Damage Repair and Regeneration Medicine of Zunyi Medical University, Zunyi, China; 3 Department of Plastic Surgery, Nanfang Hospital, Southern Medical University, Guangzhou, Guangdong China

**Keywords:** Wound healing, Stromal vascular fraction gel, Adipose stem cells

## Abstract

**Background:**

Chronic refractory wounds easily relapse and seriously affect the patients’ quality of life. Previous studies have shown that stromal vascular fraction gel (SVF-gel) significantly promotes the early healing of chronic wounds; however, the mechanisms of SVF-gel function per se remain unclear, and a long-term follow-up is lacking. This study aims to explore the mechanisms of SVF-gel promoting the healing of chronic wounds and follow up the long-term efficacy of SVF-gel.

**Methods:**

Autologous SVF-gel transplantation was performed in 20 patients with chronic wounds (from March 2016 to September 2019), and the size of the wound before and after SVF-gel transplantation was observed. The conditioned medium (CM) was harvested from SVF-gel under serum-free, serum-deprivation and 10% fetal bovine serum (FBS) microenvironment in vitro, respectively. The concentration of the growth factors in the two kinds of gel-CM was tested, and their effects on the proliferation and migration of human dermal fibroblasts (HDFs) were detected.

**Results:**

All patients had 100% wound closure eventually, and the average time to complete closure was 28.3 ± 9.7 days. The time of follow-up ranged from 2 to 6 years, and there was no wound recurrence. Interestingly, the concentrations of epidermal growth factor and transforming growth factor *β*1 of the CM were higher in serum-free and serum-deprivation condition than in 10% FBS microenvironment (*p* < 0.05). Correspondingly, the proliferation and migration ability of HDFs treated with gel-CM from serum-free condition were stronger than those treated with gel-CM from serum-deprivation (2% FBS) or 10% FBS microenvironment (*p* < 0.05).

**Conclusion:**

These results indicate that it is safe, effective, and lasting in effect to treat chronic wounds with SVF-gel and mechanisms of action that include secreting various cytokines and promoting cell proliferation and migration ability.

*Trial registration*: Chinese Clinical Trail Registry, ChiCTR2000034624. Registered 12 July 2020—Retrospectively registered, http://www.chictr.org.cn/showproj.aspx?proj=56058

**Supplementary Information:**

The online version contains supplementary material available at 10.1186/s13287-023-03389-2.

## Background

Chronic refractory wounds refer to wounds that fail to achieve timely structural and functional integrity after standard clinical treatment and thus develop into a state of chronic pathological inflammation. With the aging of the population and the improvement of living quality, the incidence of chronic wounds, which are common in diseases such as diabetes, venous stasis ulcers, pressure sores, deep burns and systemic sclerosis, has been continuously increasing [1]. Thus, the treatment of chronic wounds has become a major problem for plastic surgeons and affects the quality of life and economic burden of patients. To date, there is no satisfactory treatment method for chronic wounds.

Recently, stem cell therapy has attracted much attention in the treatment of chronic wounds by regulating wound inflammation and promoting wound vascularization and epithelization. Adipose-derived stromal vascular fraction (SVF) is a mixed cell population other than mature adipocytes in adipose tissue, including adipose-derived stem cells (ASCs)—the major components, adipose precursor cells, macrophages, endothelial cells, and pericytes [2]. ASCs are currently the most widely used type of mesenchymal stem cells due to their wide sources, rich content, and easy access. Several studies suggest that ASCs have therapeutic potential in the treatment of chronic wounds. Not only can ASCs differentiate into a variety of cells needed for wound repair, such as epidermal cells and endothelial cells, but they also have a powerful paracrine function and can secrete a variety of cytokines and growth factors to promote wound angiogenesis and epidermal regeneration [3–7]. However, the optimal cell delivery method of ASCs has still not been effectively determined [8]. Moreover, the separation of ASCs requires digestion using heterogeneous collagenases, which increases the risk of biological contamination; its cultivation and purification also require specific laboratory equipment and personnel. These factors limit the further application of ASCs in clinical practice.

The stromal vascular fraction gel (SVF-gel) is a high-concentration product rich in extracellular matrix (ECM) and SVF cells obtained through purely physical methods [9]. Its preparation does not require the use of heterogeneous collagenase digestion, which reduces the risk of biological contamination. Moreover, compared with SVF suspension, SVF-gel shows a better therapeutic effect in the treatment of wound models in nude mice [9]. Our previous studies found that conditioned medium (CM) from SVF-gel accelerates wound healing in rats [10, 11]. However, the interference of serum was not eliminated when the culture medium was obtained, while several studies have shown that serum affects the paracrine function of stem cells [12, 13]. Subsequently, our clinical cases showed that SVF-gel significantly promoted chronic wound healing by promoting angiogenesis and collagen regeneration compared with negative pressure wound treatment [14]. However, long-term efficacy observation is lacking, while chronic refractory wounds were easy to relapse.

In the present study, we explored the mechanism of SVF-gel promoting wound healing without the interference of serum and further confirmed the clinical efficacy of SVF-gel in treating chronic wounds by extending the follow-up time.

## Methods

### Subjects

This study was approved by the Ethics Committee of the Affiliated Hospital of the Zunyi Medical University and followed the principles outlined in the Declaration of Helsinki (ChiCTR2000034624). Autologous SVF-gel transplantation was performed in 20 patients with chronic wounds who were hospitalized in the Plastic Surgery Department of the Affiliated Hospital of Zunyi Medical University from March 2016 to September 2019. All subjects were provided written informed consent for participation in this research. The inclusion criteria were as follows: (1) ages of > 18 and < 80 years; (2) a nonhealing ulcer greater than 2 cm^2^; and (3) the ulcer was not healed for more than 3 months due to various reasons. Criteria for exclusion were: (1) coagulopathy or mental illness; (2) severe malnutrition; (3) participating in another clinical trial; (4) pregnancy; (5) allergy to anesthetics; and (6) critically ill patients such as those with multiple organ dysfunction, severe infection, or shock.

### Harvest of the SVF-gel

**Fig.1:**
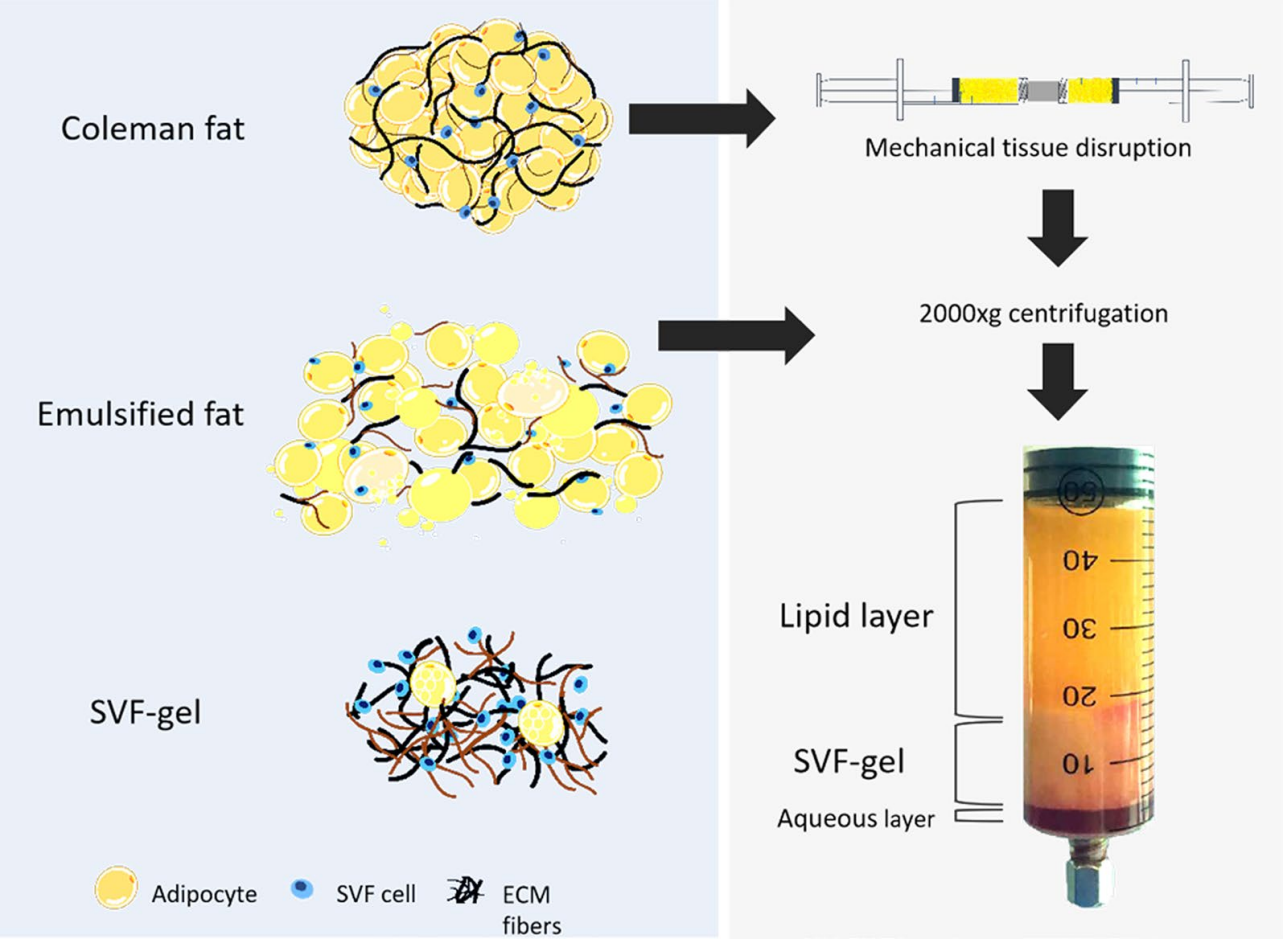
The schematic diagram of preparation and constituent for SVF-gel

The preparation of the SVF-gel was performed as previously reported [14]. Briefly, thigh liposuction was performed with a 3-mm multiport cannula, and the liquid portion was discarded by sedimentation. Then, centrifugation was performed at 1200 × g for 3 min. The dry fat was then mechanically emulsified by shifting between two 10-mL syringes connected by a female-to-female Luer Lock connector. After processing, the fat turned into an emulsion and was filtered using a Nano Transfer filter (Tulip Medical Products, San Diego, CA, USA) to remove connective tissue remnants. Next, a flocculate was observed within the emulsion. The mixture was then centrifuged at 2000 × g for 3 min and divided into three different layers: an oily layer, an SVF-gel layer, and a small amount of aqueous layer. Finally, the SVF-gel layer in the centrifuge tube was collected (Fig. 1).

### Wound intervention

Under spinal anesthesia, thigh liposuction was performed, and the SVF-gel was prepared according to the methods mentioned above. The wound area was determined by using Adobe Photoshop CS6.0 software according to the pixel/area ratio. Then, wound debridement was performed, and the prepared SVF-gel was applied. The volume of the SVF-gel used was depended on the area of the wound, and the ratio was approximately 0.25 mL/cm^2^. Approximately three-fourths of the prepared SVF-gel was directly injected into the wound bed and edge of the wound, and the remaining gel was used to cover the surface of the wound. Then, the wound was covered with sterile saline gauze. In general, the dressing was changed every 2–3 days and the wound was again enclosed with physiological saline gauze to retain moisture. As for outpatients, they returned weekly for wound inspections. Wound photographs from each subject at different treatment periods were obtained by a digital camera (Canon, Japan). The measured resolution at which an image was acquired was 72 dpi, and the resolution of each figure was adjusted to 300 dpi by using Adobe Photoshop CS6.0 software.

Time of 50% size reduction and full closure of the wound were recorded, respectively. All the patients were followed up for at least 2 years to monitor for wound recurrence. They also received the same conventional treatment for the duration of this study, including the managing of blood pressure, blood glucose, and application of antibiotics.

### Collection of the gel-CM

To obtain the gel-CM, 1 mL of the SVF-gel from the patients with chronic wounds was equally divided into two parts and inoculated in T25 culture flasks. For each culture flask, 5 mL of either standard media or serum-deprivation media was added. The standard media consisted of the Dulbecco’s modified Eagle media/F12 (DMEM/F12; Invitrogen, CA, USA) supplemented with 10% fetal bovine serum (FBS; Bovogen, Australia) and 1% penicillin–streptomycin solution (Invitrogen, USA), while the serum-deprivation media consisted of DMEM/F12 supplemented with 2% FBS and 1% penicillin–streptomycin solution. The flasks were incubated at 37 °C and a humidity of 5%. After 24 h for the suspension culture, the mixture was continuously filtered with 100-μm and 40-μm mesh filters to remove the tissue block. The filtered liquid was centrifuged at 300 × g for 5 min and then filtered with a 0.22-μm mesh to remove the debris. Ultimately, the CM was collected and stored at − 80 °C.

### Analysis of the gel-CM content

The concentrations of several cytokines, namely basic fibroblast growth factor (bFGF), epidermal growth factor (EGF), transforming growth factor (TGF-*β*1), and vascular endothelial growth factor (VEGF) in the two kinds of gel-CMs, were measured using the human enzyme-linked immunosorbent assay (ELISA) kits (NeoBioscience, China) according to the manufacturer’s instructions.

### Isolation of the SVF cells and differentiation of the ASCs

The SVF cells were separated from the SVF-gel harvested from patients with chronic wounds by collagenase digestion. Briefly, the SVF-gel was digested with phosphate-buffered saline (PBS) containing 0.075% collagenase on a shaker at 37 °C for 30 min. The mature adipocytes and connective tissues were removed by centrifugation at 800 × g for 5 min. The cell particles were resuspended and filtered through a 100-μm mesh.

The isolated SVF cells were subsequently cultured in a 5% CO_2_ control medium (DMEM containing 10% FBS, 100 U/mL penicillin, and 100 mg/mL streptomycin) at 37 °C overnight. Then, the SVF cells were cultivated for 3–5 days until fully confluent, and the cells from passages 3–5 were used. As mentioned previously [15], the ASCs cultured and induced in vitro were detected by Oil Red O, Alizarin Red S, and Alcian Blue staining to measure the adipogenic, osteogenic, and chondrogenic differentiation ability, respectively, so that to identify the multidirectional differentiation of the ASCs. And the results were observed and photographed with the inverted microscope (IX70-142, Olympus, Japan).

### Scanning electron microscopy (SEM)

Normal adipose tissue, SVF-gel, and decellularization of adipose tissues (DAT) were fixed in 0.1 M phosphate buffer with 2% glutaraldehyde, placed in the same buffer for 1 h, dehydrated with increased acetone concentration, and dried at the critical point. The colloidal silver was fixed on the short post, and the gold was sputtered with the MED010 type coating machine (Tokyo Hitachi Co., Ltd., Japan) and observed under the S-3000N scanning electron microscope (Tokyo Hitachi Co., Ltd., Japan).

### Cell viability assay

The human primary dermal fibroblast cell lines (catalog #PCS-201-012, ATCC, VA, USA) were purchased and then cultured and expanded in standard media supplemented with 10% FBS and 1% penicillin–streptomycin and used for the experiments in passages 3–5. When the fibroblasts reached 70% confluence, they were trypsinized and subcultured to culture flasks.

The viability of the HDFs after treatment with gel-CM was assessed using the Cell Counting Kit-8 (CCK-8) assay (Dojindo, Japan). The cells (1 × 10^4^/well) were plated on 96-well plates for 24 h, followed by treatment with the gel-CM from the serum-free media (gel-CM), serum-deprivation media (2% FBS), and standard media (10% FBS). After treatments for 24, 48, and 72 h, the cell viability was measured using a CCK-8 assay kit (Dojindo, Japan) according to the manufacturer’s instructions, respectively. All experiments were performed three times.

### Scratch assay

The human primary dermal fibroblast cell line (catalog #PCS-201-012, ATCC, VA, USA) was purchased and then cultured and expanded in standard media supplemented with 10% FBS and 1% penicillin–streptomycin and used for the experiments in passages 3–5. The fibroblasts were plated on six-well plates with standard media until they reached 80% confluence. A linear wound was generated using a sterile 200-μL pipette tip, and the cellular debris were washed with PBS. The culture medium was then immediately changed with the gel-CM from the serum-free media (gel-CM), serum-deprivation media (2% FBS), and standard media (10% FBS), and then, the cells were cultured for 12 and 24 h. Three representative images from each well of the scratched area were taken using a microscope (IX70-142, Olympus, Japan) to estimate the migration ability of the fibroblasts. And the cell migration rate was calculated by Image J software (National Institutes of Health, Bethesda, MD). The experiments were repeated three times.

### Statistical analysis

The normally distributed data were expressed as the mean ± standard deviation (SD), and the skewed data were expressed as the median. Comparisons between the two groups were analyzed by Student’s t-test and among more than two groups by analysis of variance (ANOVA), followed by post hoc Fisher’s least significant difference (LSD) method. *P* < 0.05 was considered significant. All analyses were performed with SPSS 19.0 (Armonk, NY).

## Results

### Clinical outcome

**Fig. 2:**
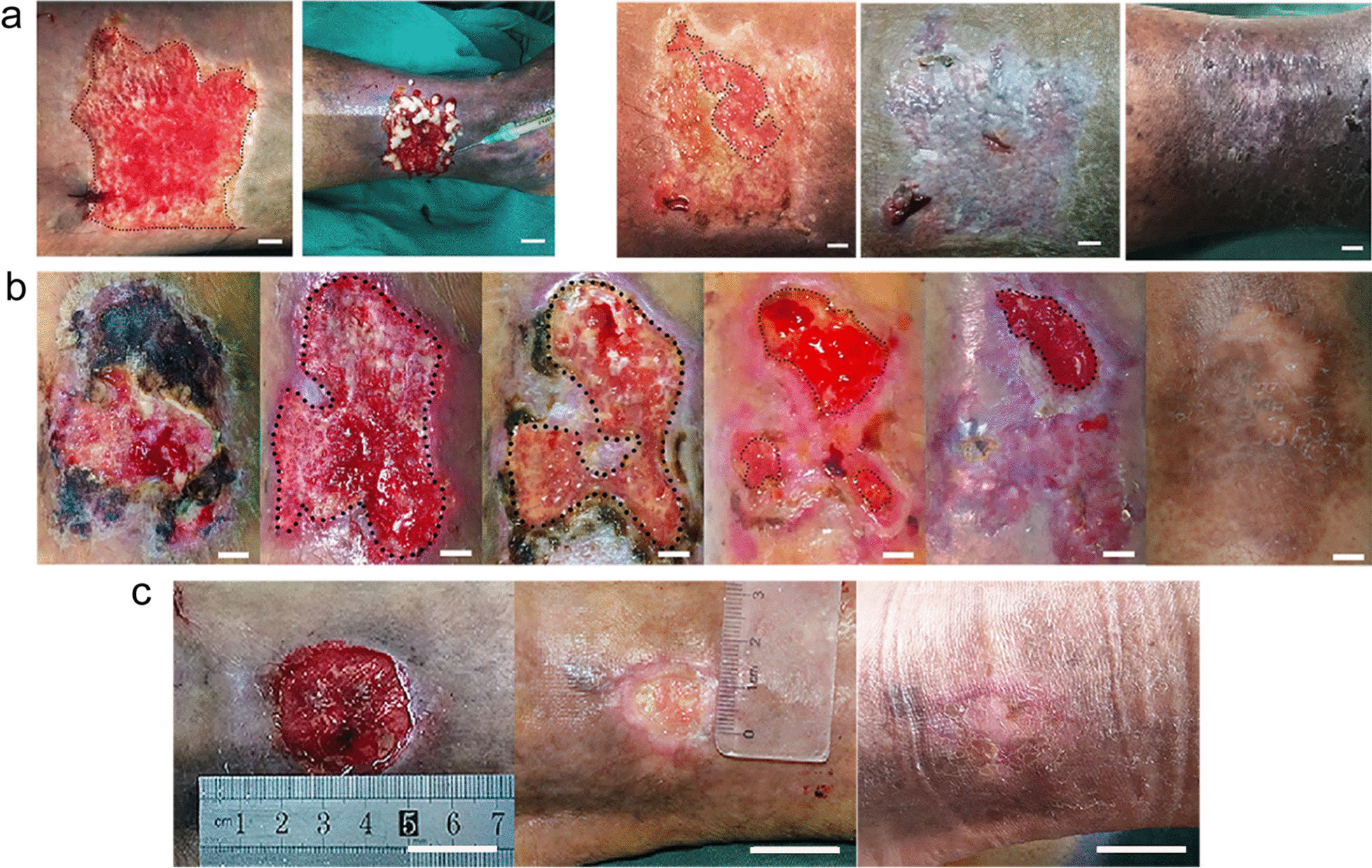
Changes in wounds after SVF-gel grafting. **a** Patient 8 was a 64-year-old male with postoperation varicose veins, hypertension, obesity, and femoral head necrosis and had an intractable chronic wound on the top of the medial malleolus. Preoperative view, after surgical debridement, original wound size was 14.60 cm^2^ (left 1). Transplantation of SVF-gel (left 2). Two weeks after the treatment of SVF-gel, the wound was mostly epithelialized. The remaining wound size was 3.06 cm^2^ (right 1). At 22 days after treatment, reepithelization of wound was completed (right 2). Three years post-surgery, the original necrosis area healed and no ulcers recurred (right 3). Scale Bar = 0.5 cm. **b** Patient 4 was a 73-year-old male with diabetes mellitus and had an intractable chronic wound on the top of lateral malleolus. Preoperative view (left 1). After surgical debridement, original wound size was 19.14  cm^2^ (left 2). Two weeks after the treatment of SVF-gel, the wound was partly epithelialized. The remaining wound size was 12.59 cm^2^ (left 3). Three weeks after treatment, the remaining wound size was 5.05 cm^2^ (right 1). Four weeks after treatment, the wound was mostly epithelialized. The remaining wound size was 2.54 cm^2^ (right 2). Six years post-surgery, the original necrosis area healed and no ulcers recurred (right 3). Scale Bar = 0.5 cm. **c** Patient 5 was a 74-year-old female with a chronic ulcer located above right medial malleolus for 5 months and had a history of great saphenous varicose vein. After surgical debridement, original wound size was 7.61 cm^2^ (left). Two weeks after treatment, the wound was mostly epithelialized. The remaining wound size was only 1.72 cm^2^ (middle). Three years post-surgery, the original necrosis area healed and no ulcers recurred (right). Scale Bar = 2 cm

There were 6 women and 14 men ranging in age from 40 to 80 years, of whom seven cases were venous stasis ulcers, three cases were diabetic ulcers, three cases were post-traumatic infection, four cases were infectious ulcers, two cases were scar ulcers, and one case was ischemic ulcer, respectively (see Additional file 1: Table S1). All wounds were unhealed for 3 months and were refractory with standard wound care. The full closure days of the wounds were 28.3 ± 9.7 days after treating with SVF-gel (Table 1). At 2 months, all the patients’ wounds were completely healed, and there was no ulcer recurrence at 2-year follow-up. Most patients were followed up for 3 years, some as long as 6 years. In addition, there were no complications at the thigh donor site where the fat was obtained. Figure 2 shows several typical cases.

**Table 1:** Summary of patient characteristics

Group	SVF-gel
Age*	59.0 ± 11.0
Female subjects	6/20
Wound size (cm^2^)#	6.55
Pre-treatment duration (months)#	5
50% size reduction (days)*	13.6 ± 4.0
Full closure (days)*	28.7 ± 9.7

*Values are expressed as mean ± standard deviation for continuous variables

^#^Values are expressed as median for continuous variables

### Cellular components of the SVF-gel

**Fig. 3:**
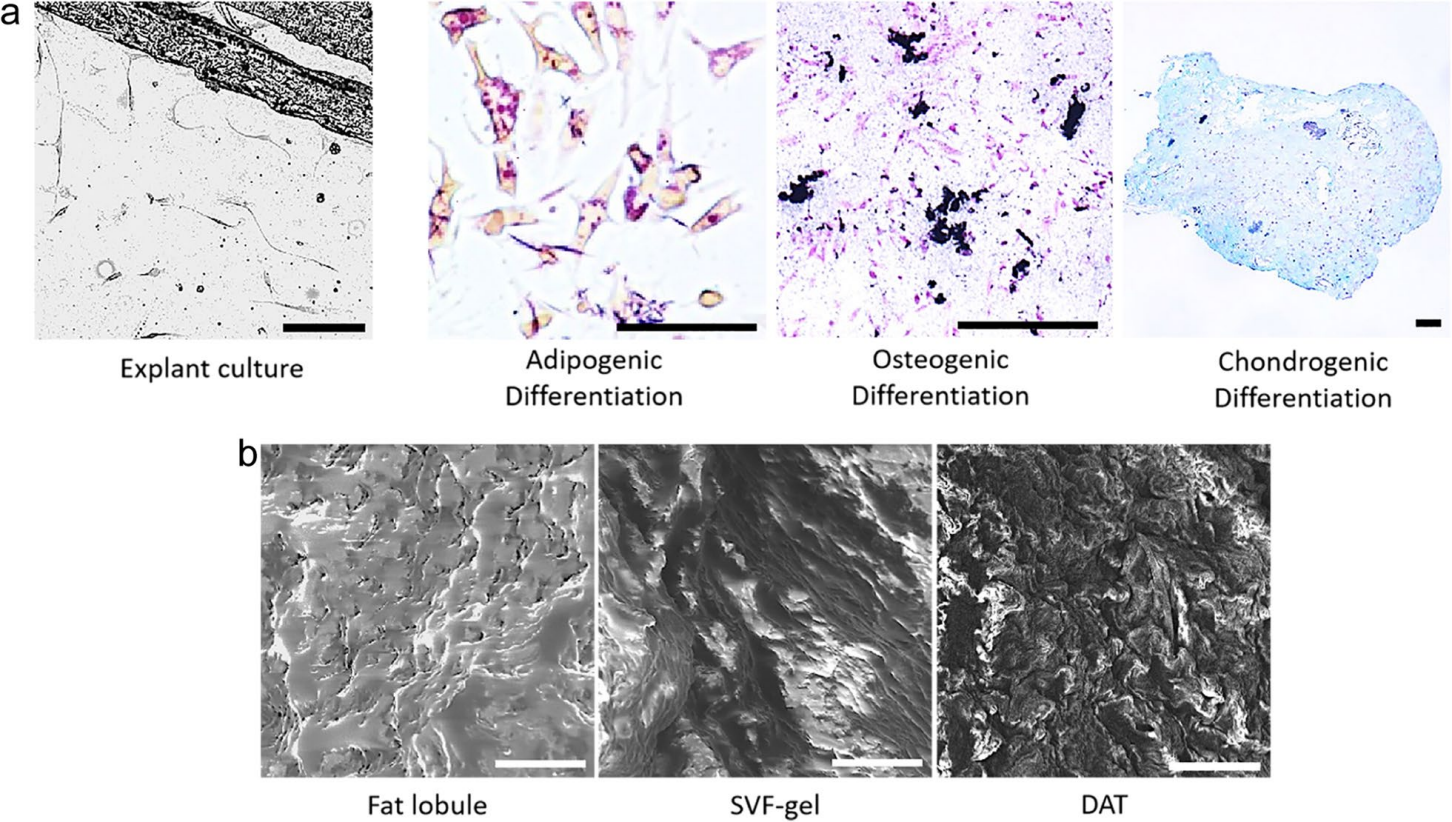
Characterization of SVF-gel. **a** Adipogenic, osteogenic, and chondrogenic differentiation of ASCs were determined by staining intracellular lipid droplets with Oil Red O, staining matrix mineralization with Alizarin red S, and staining glycosaminoglycans with Alcian blue, respectively. Scale bars = 100 μm. **b** Scanning electron microscopy showed that SVF-gel has dense and neatly arranged extracellular matrix than the normal adipose tissue and decellularized adipose tissue. Scale bars = 100 μm

The ASCs isolated from the SVF-gel showed fibroblast morphology and were easily expanded when cultured in vitro in a conventional culture medium. To verify their multipotency, the ASCs were incubated in mediums that could induce adipogenic, osteogenic, and chondrogenic differentiation, respectively. The cells were stained with Oil Red O to demonstrate the accumulation of lipid droplets in the cells. The evaluation of calcium deposition in the extracellular matrix by Alizarin Red S staining confirmed the osteogenic ability of the ASCs. The proteoglycan content was evaluated by Alcian blue staining to confirm the chondrogenesis induction of the ASCs. These results confirmed that the ASCs have proliferation and differentiation potential (Fig. 3A).

### Structural changes of the SVF-gel

Scanning electron microscopy was performed to examine the changes in the normal adipose tissue, SVF-gel, and DAT. There was no change in the extracellular matrix of the normal adipose tissue. However, SVF-gel had dense and neatly arranged extracellular matrix, while DAT had many dense and discontinuous extracellular matrix deposits (Fig. 3B).

### ELISA detection of the secreted growth factors released from the SVF-gel

**Fig. 4:**
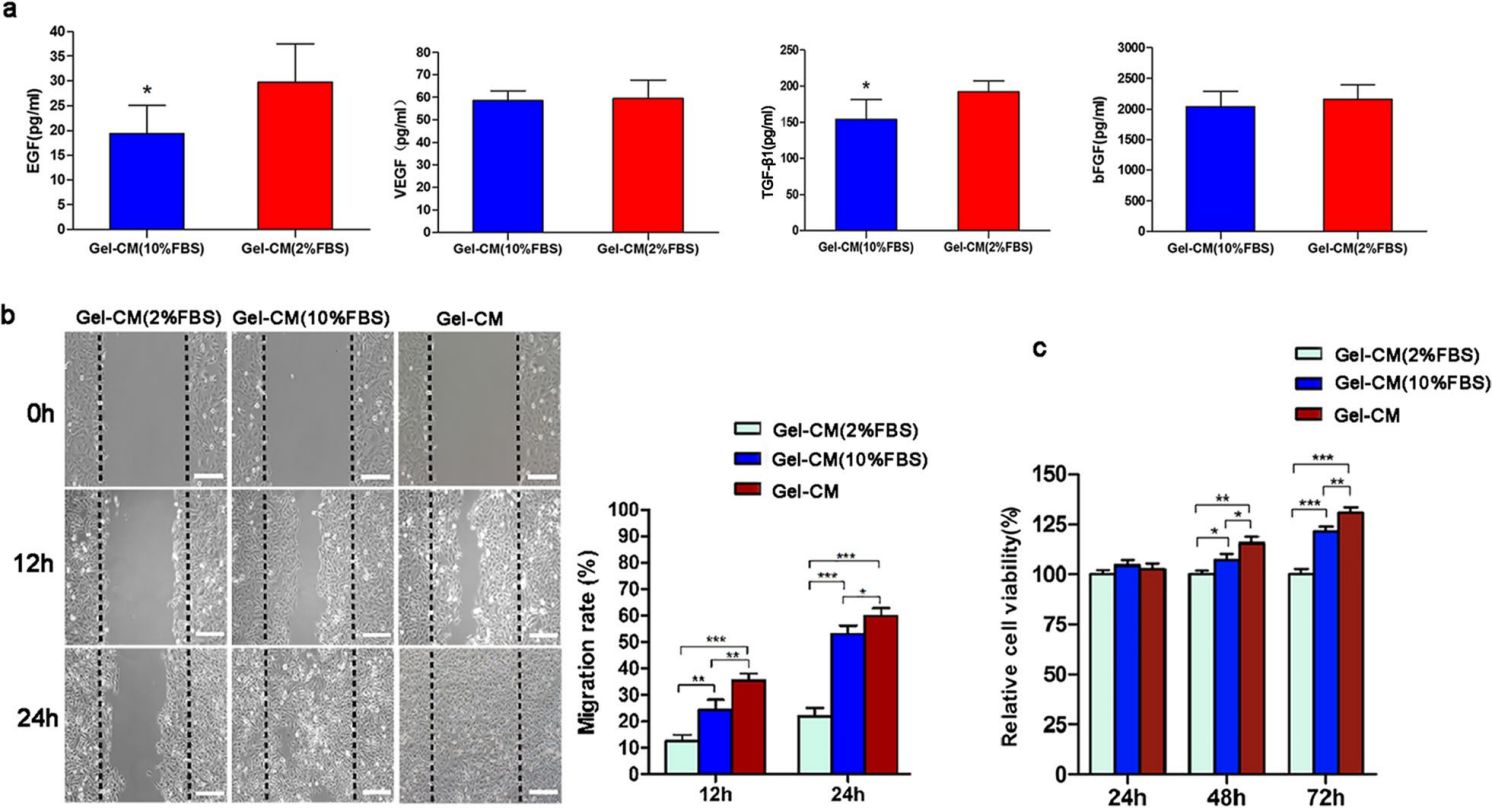
In vitro experiments. **a** Quantification of growth factor expression by ELISA. Quantification of bFGF, EGF, TGF-*β*1, and VEGF in gel-CM from serum-deprivation (2% FBS) condition and 10% FBS condition. **b** gel-CM enhances the migration of fibroblasts. Representative pictures of migrating fibroblasts in scratch-wound assay and migration rate after treatment with gel-CM from serum-free condition, serum-deprivation (2% FBS), or 10% FBS microenvironment. Scale bars = 100 μm. **c** The proliferation of fibroblasts after treatment with gel-CM from serum-free condition, serum-deprivation (2% FBS), or 10% FBS microenvironment was determined by a CCK8 assay. ^*^p < 0.05, ^**^p < 0.01,.^***^p < 0.001

Given the hypothesis that the paracrine effect of SVF-gel may have an important role in promoting wound healing, the concentrations of several growth factors secreted by the SVF-gel were tested through ELISA. The results showed that compared with that of the 10% FBS group, the gel-CM from the serum-deprived medium produced more EGF (19.40 ± 5.66 pg/mL vs. 26.27 ± 4.01 pg/mL) and TGF-*β*1 (154.20 ± 27.13 pg/mL vs. 191.94 ± 15.54 pg/mL) (*P* < 0.05), while the expression of bFGF (2,037.17 ± 254.06 pg/mL vs. 2,164.89 ± 233.66 pg/mL) and VEGF (59.56 ± 8.16 pg/mL vs. 58.58 ± 4.45 pg/mL) (*P* > 0.05) was not significantly different (Fig. 4A).

### Effect of the gel-CM on the migration and proliferation of the HDFs

The fast migration of fibroblasts is an essential step for wound healing. To further examine whether the gel-CM could promote dermal fibroblast migration, we performed the wound scratch assay (Fig. 4B). The results showed that dermal fibroblasts treated with gel-CM from serum-free medium had better migration ability and the wound was covered significantly faster at 24 h after scratching compared with that treated with standard medium or serum-deprivation medium, suggesting that the gel-CM accelerated the dermal fibroblast migration and it worked better in serum-free condition, as shown in Fig. 4B and C. Additionally, we further quantified the scratch wound area and found a statistically significant increase in the fibroblast migration rate in the gel-CM-treated group. To confirm the effect of the gel-CM on the proliferation of HDFs, we performed a CCK-8 assay. Compared with the HDFs in the serum-deprivation or 10% FBS microenvironment, no significantly increased cell viability of the HDFs was observed at 24 h after treatment with the gel-CM from the serum-free condition. However, a remarkable increased cell viability was detected at 48 h (*P* < 0.01), and the difference was much greater at 72 h (*P* < 0.001; Fig. 4C).

These results indicated that gel-CM from serum-free condition had better effect on promoting proliferation and migration of fibroblasts compared with those from serum-deprivation (2% FBS) or 10% FBS condition.

## Discussion

Although we reported in our previous paper that SVF-gel could promote chronic wound healing, only a few cases were included, and the follow-up time was too short. In our current study, more clinical cases were included and the results showed that autologous SVF-gel transplantation could significantly promote the healing of chronic wounds, with no complications and recurrences occurred even at a follow-up time as long as 6 years.

For decades, many therapeutic methods have been used to treat chronic wounds. Negative pressure wound therapy (NPWT) is one of the most common treatment methods for chronic wounds. The possible mechanisms of NPWT in the treatment of wounds include reducing matrix metalloproteinases, stimulating granulation tissue formation and angiogenesis, reducing edema, thereby improving blood flow and reducing bacterial proliferation [16–18]. It was reported that the area of chronic wounds after the application of NPWT was reduced by 17.44% per week on average [19]. Our previous study also showed that the weekly wound healing rate of negative pressure sealing treatment in the control group was 10.16% ± 2.67%, while the weekly wound healing rate in the SVF-gel group was 34.55% ± 11.18% [14]. In addition, the wound healing rate after SVF-gel transplantation was also significantly higher than that of various other ASCs therapies, including adipose tissue transplantation [20, 21], freshly isolated SVF cells [22], uncultured human adipose tissue treated cells [23], in vitro culture-expanded ASCs, and ASC-based biological dressing [24–26]. Therefore, as a novel ASCs/SVF therapy, SVF-gel has attractive clinical application prospects.

Previously, we have revealed that the infiltration of lymphocytes in the deep dermis of the patients treated with SVF-gel was significantly decreased compared with NPWT, suggesting the potential immunomodulatory effect of SVF-gel. Furthermore, Masson staining showed more collagen production, and CD31 immunohistochemistry also showed increased endothelial-specific CD31 expression in wounds in SVF-gel group, which indicated that SVF-gel treatment promoted more angiogenesis and better tissue regeneration [14]. And in our current study, we illustrated that SVF-gel also contributed to chronic wound healing by promoting the migration and proliferation of HDFs, which further enriched the mechanisms of SVF promoting wound healing. However, more detailed data and further investigation are still needed to explore the complex effect of SVF-gel in the future.

According to our previous research, the density of ASCs and endothelial cells (ECs) in SVF-gel was 1.9 ± 0.2 × 10^5^ cell/mL and 7.7 ± 2.4 × 10^4^ cell/mL, respectively. Many studies have shown that ASCs can promote the regeneration and migration of epithelial cells and fibroblasts, increase angiogenesis, and play an immunomodulatory role by secreting a variety of growth factors, such as VEGF, HGF, EGF, and FGF [27–29]. ECs directly form capillaries and are the most important functional cells in angiogenesis [30]. Therefore, SVF cells (including ASCs, ECs, and pericytes) may play an important role in SVF-gel promoting the healing of chronic wounds and ensuring their long-lasting efficacy. In addition, in this study, the SEM showed that the SVF-gel had a large number of concentrated and neatly arranged ECM, which mainly included collagen, elastin, mucopolysaccharide, and fibronectin. Studies have found that collagen and fibronectin are important components in the process of wound healing [31]. In addition, ECM can also promote angiogenesis [32], which may explain the increased new blood vessels seen on the histology done after SVF-gel transplantation in our previous study [14]. This may also be the another factor involved in the promotion of chronic wound healing by SVF-gel.

Recently, accumulating studies have revealed that the beneficial effects of stem cell therapy result from their ability to secrete bioactive factors that exert a positive impact on the recipient cells, rather than their capability to differentiate into the required cells [33–36]. It has been well established that ASCs-CM possesses anti-inflammatory and immunomodulatory activities and is able to ameliorate allergic airway inflammation and induce immunologic tolerance [37, 38]. Additionally, it has been reported that numerous neurotrophic, neurogenic, axon guidance, and axon growth proteins were detected within the secretion or extract of ASCs, which are beneficial to the recovery of patients with neurological diseases such as stroke and epilepsy [39–41]. Moreover, it is worth mentioning that some clinical studies showed that ASCs-CM had a positive effect on accelerating wound healing after fractional carbon dioxide laser resurfacing and combined with that resurfacing for atrophic acne scars and skin rejuvenation [42, 43]. Therefore, the above-mentioned data suggest that the ASCs secretome [44], including a wide range of bioactive molecules like cytokines, chemokines, growth factors, and exosomes, has a beneficial effect on different pathophysiological status and disease.

Chronic wound is always accompanied by persistent tissue ischemia and hypoxia, leading to insufficient nutrient supply to the cells. Furthermore, previous study showed that the concentration of serum may affect the effect of gel-CM [45]. To simulate the chronic wound environment with insufficient nutrient supply, different concentrations of serum were used in the current study to treat SVF-gel to obtain different gel-CMs. The results showed that the secretion of the growth factors, especially the EGF and TGF-*β*1, within the gel-CM increased in the serum-reduced medium, wherein only 2% serum was added. In addition, the CCK-8 and scratch test also showed that the gel-CM from the serum-free condition significantly promoted the proliferation and migration of HDFs compared with that in the serum-deprivation (2% FBS) or 10% FBS microenvironments. Our results were consistent with those of previously published studies, wherein ASCs-CM could accelerate the proliferation and migration of a variety of cells, including ASCs, HDF, ECs, and keratinocytes [46–50]. These positive effects may be due to the enhanced expression of the endogenic genes from various cells within the SVF-gel as lower serum concentration was reported to induce the adaptation of the ASCs to a low growth factor environment by increasing the endogenous production of mitotic agents [51]. In addition, under serum starvation conditions, ASCs can simulate limited blood supply, thereby simulating hypoxic and ischemic conditions in the body and then secreting more growth factors or cytokines. This may also be the reason why low serum can stimulate the SVF-gel to secrete more growth factors such as bFGF, EGF, VEGF, and TGF-*β*1. Thus, we suggested that chronic wounds provide SVF-gel with a low-serum (low-nutrients) environment that stimulates SVF-gel to secrete more growth factors to promote wound healing.

Wound healing is a dynamic and complex process that not only requires careful and coordinated interactions between cytokines, resident cells, and infiltrating cells [52], but also requires an adequate blood supply. Among these growth factors and cytokines, EGF can accelerate re-epithelialization by increasing the proliferation and cell migration of keratinocytes [53, 54], while bFGF and VEGF mainly promote fibroblast proliferation and migration [55, 56]. In addition, it is reported that TGF-*β*1 plays an active role in skin wound healing by promoting epithelial regeneration, wound contracture, angiogenesis, scar formation and ECM deposition [56]. In particular, TGF-*β*1 stimulates the migration of dermal fibroblasts and keratinocytes more obviously [57, 58]. In our study, the promotion of SVF-gel in chronic wound healing may be explained as a serum starvation condition constructed by chronic wounds encourages SVF-gel to secret more cytokines or growth factors required for wound healing.

Generally, there are some merits of SVF-gel as a novel stem cell therapy as compared to other traditional ASC therapy. SVF cells are still protected by ECM that is three-dimensional and has natural scaffolds, which may improve the long-term retention rate of SVF cells as compared to those of ASCs or SVF suspensions. In addition, SVF cells avoid the usage of exogenous products, the application of collagenase, and in vitro culture process as compared to ASC therapy. Moreover, SVF-gel can be harvested quickly in the operating room, and a specialized laboratory is not required. The clinical results showed that autologous SVF-gel transplantation significantly promoted chronic wound healing, with no complications and recurrence of ulcers. However, the optimal volume, time, and duration of SVF-gel transplantation need to be further determined. In addition, a larger-scale, multicenter clinical controlled trial is still needed in the future.

## Conclusions

In conclusion, our research showed that autologous SVF-gel transplantation was not only safe and effective in promoting chronic wound healing, but also had a long-lasting effect. The reason may be that chronic wounds provide SVF-gel with a low-nutrients environment that stimulates SVF-gel to secrete more growth factors needed for the proliferation and migration of cells playing critical roles in wound healing.

## Supplementary Information

**Additional file 1: Table S1**. General condition of the patient.

## Data Availability

The datasets generated during and/or analyzed during the current study are available from the corresponding author on reasonable request.

## Abbreviations

SVF-gel: Stromal vascular fraction gel
CM: Conditioned medium
FBS: Fetal bovine serum
HDFs: Human dermal fibroblasts
EGF: Epidermal growth factor
TGF-*β*1: Transforming growth factor *β*1
ASCs: Adipose stem cells
ECM: Extracellular matrix
DMEM/F12: Dulbecco’s modified Eagle media/F12
bFGF: Basic fibroblast growth factor
VEGF: Vascular endothelial growth factor
ELISA: Enzyme-linked immunosorbent assay
PBS: Phosphate-buffered saline
DAT: Decellularization of adipose tissues
CCK-8: Cell counting kit-8
SD: Standard deviation
ECs: Endothelial cells
