# Supplementary material for: Long-term follow-up and exploration of the mechanism of stromal vascular fraction gel in chronic wounds
Source: Stem Cell Res Ther. 2023 Jun 19;14:163. doi: 10.1186/s13287-023-03389-2 (PMC10280847; doi:10.1186/s13287-023-03389-2)
Supplement: Supplementary file 1 — Additional file 1: Table S1. General condition of the patient. [file 13287_2023_3389_MOESM1_ESM.docx]

Supplementary table 1 General condition of the patient.

| **Patient** | **Age** | **Sex** | **Wound Pathophysiology** | **Duration (months)** | **Wound size (cm^2^)** | **Wagner grade** | **50% size reduction (days)** | **Full closure (days)** | **Follow-up time(months)** |
| --- | --- | --- | --- | --- | --- | --- | --- | --- | --- |
| SVF1 | 67 | M | Venous ulcer, post-traumatic infection | 3 | 6 | - | 19 | 34 | 36 |
| SVF2 | 67 | M | Infection, hyperglycemia | 3 | 2.3 | - | 10 | 15 | 36 |
| SVF3 | 50 | M | Infection; Surgical history of varicose veins | 60 | 5.7 | - | 12 | 19 | 36 |
| SVF4 | 73 | M | Post-traumatic infection, Diabetes | 5 | 19.14 | 2 | 18 | 30 | 58 |
| SVF5 | 74 | F | Venous ulcer | 5 | 7.6 | - | 16 | 31 | 57 |
| SVF6 | 40 | M | Venous ulcer | 12 | 6.9 | - | 13 | 25 | 36 |
| SVF7 | 42 | F | Diabetic ulcer, post-traumatic infection | 3 | 9.4 | 2 | 22 | 52 | 36 |
| SVF8 | 64 | M | Venous ulcer,hyperglycemia | 14 | 14.60 | - | 14 | 35 | 72 |
| SVF9 | 51 | M | Deep burn | 3 | 3.5 | - | 10 | 21 | 36 |
| SVF10 | 53 | M | Venous ulcer,History of flap grafting | 12 | 11.03 | - | 14 | 31 | 36 |
| SVF11 | 52 | F | Post-traumatic infection,obesity | 4 | 5.8 | - | 12 | 28 | 36 |
| SVF12 | 68 | M | Post-traumatic infection | 3 | 5.5 | - | 10 | 23 | 36 |
| SVF13 | 66 | M | Venous ulcer, Deep vein thrombosis | 6 | 4.14 | - | 5 | 14 | 36 |
| SVF14 | 53 | M | Multiple skin ulcers | 3 | 4.65 | - | 17 | 35 | 36 |
| SVF15 | 60 | F | Ulcer,Infection | 3 | 7.82 | - | 10 | 25 | 24 |
| SVF16 | 63 | M | Venous ulcer, hypoproteinemia | 13 | 22.84 | - | 14 | 32 | 30 |
| SVF17 | 80 | F | Ischemic ulcer | 8 | 11.52 | - | 18 | 42 | 40 |
| SVF18 | 46 | F | Diabetic foot | 9 | 8.96 | 2 | 14 | 39 | 35 |
| SVF19 | 55 | M | Scar ulcer, Burn scar | 4 | 6.2 | - | 10 | 16 | 25 |
| SVF20 | 55 | M | Diabetic foot | 6 | 3 | 2 | 13 | 27 | 27 |
